# Chromogenic medium versus PCR–RFLP in the speciation of Candida: a comparative study

**DOI:** 10.1186/s13104-019-4710-5

**Published:** 2019-10-22

**Authors:** Sankar Leena Sankari, Krishnan Mahalakshmi, Venkatesan Naveen Kumar

**Affiliations:** 1 Department of Oral Pathology and Microbiology, Sree Balaji Dental College and Hospital, Bharath Institute of Higher Education and Research, Velachery-Tambaram Road, Chennai, 600100 India; 2 Department of Microbiology/Research Lab for Oral‑Systemic Health, Sree Balaji Dental College and Hospital, Bharath Institute of Higher Education and Research, Velachery-Tambaram Road, Chennai, Tamilnadu 600100 India; 3 ImmuGenix Biosciences Pvt Ltd, No 16/2 Nattal Garden 1st Street, Perambur, Chennai, 600011 Tamil Nadu India

**Keywords:** Candida, HiCrome Candida differential agar, PCR–RFLP

## Abstract

**Objective:**

*Candida* species is implicated in a wide array of clinical infections. Speciation of Candida strains is of prime importance in the epidemiological survey and laboratory diagnosis as there is an upswing of antifungal resistance and changing trends in the antifungal resistance pattern among *C. albicans* and non *albicans Candida*. Varied phenotypic methods are available for the identification of *Candida* species which vary in principles and cost factors. Chromogenic agar medium (HiCrome *Candida* differential agar) is one of the preferred phenotypic methods in limited resource laboratories. Hence, this study was aimed to assess the reliability of HiCrome *Candida* differential agar, M1297A (HiMedia) in the identification of *Candida* species compared polymerase chain reaction–restriction fragment length polymorphism (PCR–RFLP). Oral *Candida* isolates (n = 194) were inoculated onto HiCrome *Candida* differential agar and the potential of *Candida* differential Agar was compared with PCR–RFLP.

**Results:**

The results were not in agreement with PCR–RFLP. Percentage of disagreement was 40.2, 50.0, 100.0 and 25.0 for *Candida albicans*, *Candida krusei, Candida glabrata* and *Candida tropicalis* respectively. PCR–RFLP demonstrated a very high discriminatory power in the identification of *Candida* species compared to agar.

## Introduction

Identification of Candida strains to the species level is increasingly necessary because of their variation both in their ability to cause infection and also in their susceptibility to antifungal agents. Species-level of yeast identification is mandatory for epidemiological purpose and laboratory diagnosis. A large variety of phenotypic methods for identification of *Candida* spp. are available which vary in principles and cost factors. As phenotypic methods demand more time and labour, a chromogenic substrate containing culture media have been used in research and clinical laboratories for the identification of *Candida* species [1]. The chromogenic media helps in identifying microbial colonies based on the colours produced due to chromogenic substrates that react with enzymes differentially secreted by microorganisms [1]. Molecular methods have high discriminatory power and hence more reliable for the identification of species [2]. This study aimed to evaluate the performance and reliability of HiCrome Candida differential Agar, M1297A (HiMedia, Mumbai, India) for the identification of *Candida* species. The potential of the chromogenic media was assessed by comparing with an economical, quick and consistent PCR–RFLP system.

## Main text

### Methods

A single colony of oral clinical *Candida* isolates (n = 194) from Sabouraud Dextrose Agar (SDA) (HiMedia, Mumbai, India) plate were inoculated onto HiCrome Candida differential agar and incubated at 37 °C aerobically for 24 h. The speciation of Candida isolates was based on the colony colour as per the manufacturer’s instructions (Table 1). Four to six isolates were inoculated per plate.

**Table 1:** Disagreement in speciation of *Candida* isolates by HiCrome agar and PCR RFLP

Candida species	Colour on HiCrome media as per manufacturer’s instruction/(no of isolates showing the respective colony colour)	PCR RFLP results consistent with HiCrome agar	PCR-RFLP disagreement with HiCrome agar
*C. albicans*	Light green (132)	79 (59.8%)	53 (40.2%)
*C. krusei*	Purple (38)	19 (50%)	19 (50.0%)
*C. glabrata*	Cream to white (6)	0 (0%)	6 (100%)
*C. tropicalis*	Blue to metallic blue (20)	15 (75%)	5 (25.0%)

DNA was extracted from all the Candida isolates (n = 194) by boiling lysis method [3]. Briefly, a single colony from fresh culture of each Candida isolate on SDA plate was inoculated into 200 µl of sterile PCR grade water and incubated in a heat block (Rivotek, India) at 100 °C for 10 min. Following incubation the sterile PCR grade water containing the DNA was immediately cooled to − 20 °C for 10 min, then centrifuged at 10,000 rpm for 5 min. The supernatant collected was used for PCR assay. PCR targeting ITS1-5.8SrDNA-ITS2 region was performed for all the Candida isolates. The 25 µl reaction volume consisted of 10 pM of Candida-ITS-primers as described by Mohammadi et al. [4] ITS1 (5′-TCCGTAGGTGAACCTGCGG-3′) and ITS4 (5′-TCCTCCGCTTATTGATATGC-3′), 2.5 µl of 10× PCR buffer with MgCl_2_, 0.4 mM of dNTP mix, 1 unit of Taq polymerase, 2 µl of DNA template. The PCR amplification was performed in Veriti 96 Thermal Cycler (Applied Biosystems, USA) with initial denaturation at 94 °C for 3 min followed by 40 cycles at 94 °C for 20 s, 55 °C for 30 s and 72 °C for 45 s, and subsequently final extension at 72 °C for 5 min. The PCR amplicons were resolved along with DNA markers in 1% agarose with ethidium bromide (0.5 µg/ml) by gel electrophoresis for 25 min at 135 V using Mupid-exU system (Takara, Japan). The gel was analysed by BioGlow UV Transilluminators (Crystal Technology, USA). To speciate Candida isolates, 8.8 µl of each ITS PCR product was digested with 0.2 µl MspI (4U) restriction enzyme (New England Biolabs) along with 1 µl of 10× Enzyme Buffer [4]. The restriction digestion was carried out in Veriti 96 Thermal Cycler (Applied Biosystems, USA), by incubating the mix at 37 °C for 60 min followed by heat inactivation at 85 °C for 5 min. The ITS PCR–RFLP products were resolved by electrophoresis on 2% agarose gel with 0.5 µg/ml ethidium bromide and restriction patterns were documented and compared with in silico restriction pattern by pDRAW32 (V 1.1.140) using sequences from NCBI.

### Results

**Fig. 1:**
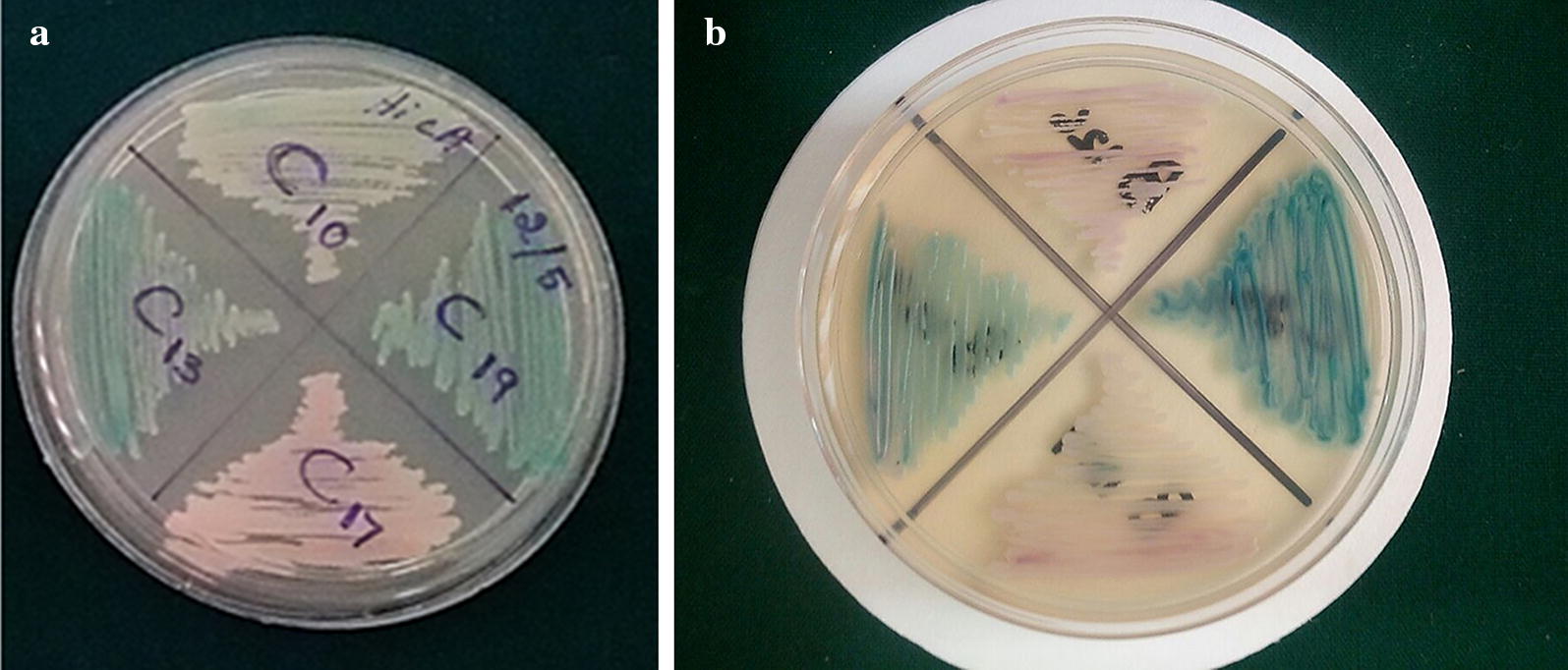
**a**, **b** HiCrome Agar plates showing different colours for identification of *Candida* species

Percent agreement was determined by the number of isolates positive by HiCrome Candida differential Agar/number of PCR–RFLP positive isolates × 100. Percent disagreement was derived by subtracting percent agreement from 100. Among the 194 candida isolates screened, 132 were identified as *C. albicans*, 36 as *C. krusei*, 6 as *C. glabrata* and 20 as *C. tropicalis* based on colour code on HiCrome agar (Fig. 1a, b). All the isolates were further identified genotypically by PCR–RFLP method. All Candida isolates identified as *C. albicans* based on colour (light green) by HiCrome agar were not in agreement with PCR–RFLP method as shown in Table 1. Similarly, the identification of three non albicans *Candida* species (*C. krusei, C. glabrata* and *C. tropicalis*) by colour code on HiCrome agar also showed a discrepancy with PCR–RFLP (Table 1).

### Discussion

The results of the present study revealed that HiCrome Candida differential Agar method of speciation is unreliable compared to PCR–RFLP. The results of this study are not in concurrence with prior studies [5–8]. The colour codes mentioned by the manufacturers on HiCrome Candida differential agar for *C. albicans*, *C. krusei*, *C. glabrata* and *C. tropicalis* were also shown by other species. This may be due to the production of similar enzymes by different species of *Candida*. The enzyme–substrate reaction was not unique to each species of *Candida*. Similar colour was produced by more than one species and hence, chromogenic media was not able to identify the species as mentioned by the manufacturer’s instruction. Genotypic methods are potentially more sensitive and reliable means for the identification of yeasts. DNA amplification with universal fungal primers followed by detection using species-specific probes greatly enhances the sensitivity of *Candida* detection [9]. Time taken by PCR–RFLP is similar to routine phenotypic conventional methods [2] but then PCR–RFLP method is highly sensitive in identifying all species of *Candida*. The sensitivity of PCR–RFLP was found to be 100% compared to HiCrome Candida differential agar [4, 10]. To conclude, PCR–RFLP method is more reliable for identifying *Candida* species than HiCrome Candida differential agar even though it may be a preferred method in a resource—limited lab setting. Hence, a molecular technique with more discriminatory power and expeditious like PCR–RFLP can be strongly recommended in the identification of *Candida* species.

## Limitations

- Different types of chromogenic media were not compared in the present study.
- All *Candida* species cannot be identified by chromogenic media.

## Data Availability

The research data are available in the main document.

## Abbreviations

PCR–RFLP: polymerase chain reaction-restriction fragment length polymorphism
ITS: internal transcribed spacer
NCBI: National Center for Biotechnology Information
